# Abstracts and Gleanings

**Published:** 1885-04-20

**Authors:** 


### ABSTRACTS AND GLEANINGS.

**Cocaine in Sleeplessness.**—Case 1. A man, aged 33, suffering from aortic disease and albuminuria, had been troubled with insomnia for a fortnight. Three minims of a 4 per cent. solution of hydrochlorate of cocaine (equal to $\frac{1}{8}$ of a grain) were administered hypodermically. The patient remarked that "he slept better than he had done for a long time." The following night one drachm of valoid of coca (a liquid extract, each drachm of which represents an equivalent quantity of the pure drug) was administered. The man did not sleep well. Two drachms of the valoid of coca were then given, and sleep was induced. The patient has continued to take this dose nightly for the past three weeks, the sleep being natural and undisturbed.

Case 2. Two drachms of the valoid were given with an equally beneficial result to a patient convalescent from an empyema, and suffering from sleeplessness.

Case 3. A woman, with tertiary syphilis, was kept awake by pain in a large rupial sore on the thigh. Two minims of the 4 per cent. solution of the hydrochlorate of cocaine, dropped on the ulcerating surface, relieved the pain, and the patient slept.

To show that coca and cocaine have no toxic action—at all events, in moderate doses—it may be worth mentioning that, in a case of rheumatism, the dose of hydrochlorate of cocaine was gradually increased to 6 minims ($\frac{1}{4}$ of a grain) hypodermically, and the valoid increased to five drachms, without any bad effect.—*Brit. Med. Jour.*

**The External Use of Chloroform in Labor.**—Dr. Svanberg's method of the external use of chloroform in labor is simple: A piece of flannel, saturated with a solution of chloroform and sweet oil, equal parts, is applied to the skin, between the umbilicus and symphysis. Light strokes over the cloth secure even distribution of the solution, and exact approximation of the fabric. The application may be renewed according to necessity. Dr. Svanberg has observed that, usually, in from five to ten minutes the chloroform has performed its function.

The indications for the external use of chloroform in labor include a variety of conditions. In general terms it may be said that the local application is designed to supersede the inhalation of chloroform.

Dr. Svanberg has successfully employed this method in cases of retained placenta with tetanus uteri, transverse presentations, with rigidly contracted uterus and escape of the liquor amnii, and breech presentations with rigidity of the internal os. In certain of these cases the inhalation of chloroform was not sufficient to relax the uterine contractions.

It is not unfitting, in this connection, to hear Dr. Svanberg's individual testimony as to the efficiency of the method: "Since that day (December, 1887) I have never used chloroform by inhalation for rigid contractions of the uterus."—*Chicago Med. Jour. and Ex.*

**The Treatment of Intra-Uterine Diseases.**—Dr. Lombe Atthill (Brit. Med. Journal, November 20, 1884) presents an able paper on this subject, and sums up his views and experiments thus:

1. Carbolic acid, in the proportion of one part of spirit to two parts of the acid, is the safest and most generally useful of all the agents employed.

2. Carbolic acid should always be applied by means of a probe, round the point of which a layer of cotton is rolled, the cotton being carried up to the fundus of the uterus at least twice on each occasion that the applications are made, which should be on every third or fourth day till marked improvement takes place.

3. Carbolic acid should never be injected into the uterus, except when combined with iodine, in the form known as iodised phenol.

4. In many cases, iodised phenol may, with advantage, be applied by means of a probe.

5. In cases in which metrorrhagia or profuse menstruation occurs, depending on an unhealthy condition of the intra-uterine mucous membrane, the cavity being dilated and the uterus enlarged, from half a drachm to a drachm of iodised phenol may be injected with great advantage.

6. In cases in which epithelioma attacks the mucous membrane of the cavity, the injection of iodised phenol promises better results than any other treatment.

7. The success likely to follow the injection of iodised phenol renders the dilation of the uterus, the use of the curette and the subsequent application of fuming nitric acid less frequently necessary than has been the case hitherto.

8. The injection of iodised phenol requires to be carried out with so much care that it should never be injected except by means of a syringe which will not contain more than a drachm.

9. The use of the fuming nitric acid should be limited, as a rule, to those cases in which dilatation has been practiced, and it should always be carried on through a tube, inserted into the cervix uteri for the purpose of protecting the sides of that canal from the action of the acid.

10. The pain produced by the application of any medical agent to the intra-uterine cavity does not bear any relation to the activity of the agent, but is due to one of two causes—either to hyperæsthesia or to a narrowness of the cervical canal, especially of the os internum.—*Detroit Lancet.*

**Calomel in the Treatment of Otorrhœa.**—Dr. J. Gottstein, in the Archives of Otology, September to December, 1884, strongly recommends the use of calomel in the treatment of otorrhœa. He says: "During the past year I have used the calomel by way of trial in a number of cases that seemed suitable, especially such as could be submitted to daily observation.

I have satisfied myself (1) that the remedy is absolutely free from irritation to the mucous membrane of the middle ear; (2) that it forms neither upon nor in the mucous membrane any precipitate difficult of removal; (3) that surprising results are often obtained under its use.

Accordingly, since the beginning of the present year, I have, in my private practice as well as in my polyclinic, employed calomel in the treatment of all cases of otorrhœa in which, following Bezold's direction, I had previously made use of boric acid, alone or as a supplementary means. I withheld the calomel only from such patients as, coming from a distance, I had an opportunity to see but once.

My observations now exceed eighty in number, so that I feel justified in communicating the results of my experience with this method.

My method of procedure is as follows: The ear is, in the usual way, syringed carefully with a weak sublimate solution (one-tenth per cent.); the residue of the secretion is forced into the external meatus by the employment of Palitzer's method, and then removed by syringing, and finally the ear is well dried with cotton.

The calomel (vapore parat.) is then blown in through a powder-blower and the auditory canal closed as well as possible by means of cotton. The further treatment is the same as with the boric acid. That on which I lay the most stress is, that calomel, in my opinion, has a much more decided and certain antiseptic action than the boric acid.

I am anxious to avoid the error into which those writers fall who overestimate the value of the remedies recommended by them. Calomel also fails in some of the cases in which powerful antiseptic action is desired, because considerable tissue-alterations in the drum-cavity are absent. Yet I have, with no method of treatment, not even with the boric acid, attained such speedy results as I have with this remedy in acute, as well as in chronic forms of otorrhœa.

The calomel is also suitable, as is the boric acid, for employment after operations in the middle ear, cauterization with nitrate of silver, the use of the galvano-cautery, and in conjunction with the alcohol treatment. In these cases, the powerful antiseptic action of the remedy is conspicuous.—*Medical News*.

**Hypodermic Injections of Morphia in Convulsions.**— Dr. Clark [Boston Med. Jour.], of Oswego, New York, reports the case of a girl eighteen months old, who had been in almost unremittent convulsions for two hours, various remedies producing no effect. The cause of the attack could not be ascertained, but was supposed to be of cerebral origin. One-sixth of a grain of morphia sulphate was injected into the arm, and in about two minutes the convulsions ceased entirely, and after a quiet sleep of three or four hours the child awoke apparently well. Twenty-four hours later, after some evidence of the attack returning, the symptoms readily subsided to doses of Dover's powder, in moderate amount, the dose not being stated. In some days, however, the child was found to have her faculty of speech, which was considerably advanced, impaired; since then her appearance has been bright, and her power of speech is constantly improving. Clark believes, from his experience, that a child of from two to five years old will stand as much opium, in proportion to the development at that age of the energies of the nervous system, as an adult. He also states that he would unhesitatingly inject a thirty-second of a grain in persistent or recurrent convulsions, and repeat in fifteen minutes if necessary. In connection with Clark's cases, Dr. Hughson reports the case of a negro boy, two years of age, who had been sick with a high fever for two days, and who had been in convulsions for an hour. The child was unable to swallow, and was rapidly sinking into a dying condition. A subcutaneous injection of a fifteenth part of a grain of the sulphate of morphia was given, and in ten minutes he was quiet, and in twenty minutes asleep; the child had malarial fever.

**A Case of Gastric Ulcer.**—Dr. Osman B. Campbell reports the following case in the College and Clinical Record, January 1, 1885:

"I was called, on March 15, 1884, to see Miss R., aged twenty-two, who was suffering with gastric pain. She had had two attacks of hæmatemesis, and was considerably prostrated from pain and inability to digest a sufficient quantity of food. I found that her trouble dated back four months, when she had had an attack of intermittent fever. Quinia and calomel were given, which broke up the fever, but she did not regain her health. She complained of a feeling of weight and soreness in the right side, near the region of the liver; her abdomen enlarged considerably, so that her clothes had to be widened to the extent of three or four inches; she was constipated and had throbbing sensations about the umbilicus. She was jaundiced, and had frequent attacks of vomiting, when the contents of the stomach would be ejected, followed by bile. These conditions continued about two months and a half, when, upon vomiting, some black, partially digested blood was ejected. She noticed the same day that her stools were black and tarry. From that time the taking of food would cause gastralgia and vomiting about twenty minutes after eating.

"Six weeks later she came under my observation. I found considerable enlargement of the liver and spleen, some bloating of the abdomen, gastralgia and vomiting, excited by taking food. She had just vomited some blood, which I found to be partially digested. She complained of a fixed pain immediately under the ensiform appendix, extending through to the spine. I diagnosticated the case as a gastric ulcer, a result of portal congestion. I restricted her diet to skim milk, diluted with one-fourth lime-water, from three to four ounces to be given every three hours, and gave a purge of calomel to relieve the portal circulation. I prescribed five-drop doses of liquor potassii arsenitis, three times a day, and gave twenty grains of quinia during the 24 hours.

"My patient began to improve immediately. There was no more gastralgia nor vomiting, nor was there any appearance of blood in the stools. Six months after I began treating the case all signs of the disease had vanished. The cause of the ulcer was, I believe, portal congestion, caused by malaria."—*Med. and Surg. Rep.*

**Cocaine in Minor Surgery.**—Dr. Irving D. Wiltnut contributes to the literature of this drug by writing as follows in the Northwestern Lancet, February 15, 1885:

I proposed to extirpate a fatty tumor the size of a hen's egg, situated near the ankle joint, a little above the outer malleolus. My patient consented to the use of cocaine. I accordingly injected fifteen drops of a four-per cent. solution into the body of the tumor. After waiting fifty seconds I made my incision over the tumor, from above downward. I then separated, with some difficulty, the skin and cellular tissue from the organized mass, and, in my attempt to sever its connection with the tissues, I was obliged to use the knife quite freely. There was little bleeding—soon controlled by the free use of cold water. The several parts were properly adjusted; stitched; a compress and a roller bandage completed the operation and dressing.

During the whole of this operation my patient looked on, an interested spectator, but confessed no pain. The incision with the knife, as well as the force required to separate the tissues from the tumor, and the final force required to separate the mass from the living tissues did not cause pain—no, not even a sensation.

The usual sick stomach followed the operation, but this cannot be ascribed to the use of cocaine.

My patient was a man of intelligence and of unquestionabie integrity, so that, as regards the total absence of pain, I do not regard I have been deceived any.—*Med. and Surg. Rep.*

**Treatment of Gonorrhœa.**—Dr. J. W. Lilly, of Pomeroy, Ohio, having been repeatedly disappointed, as we all are, in the treatment of gonorrhœa, cast about him for some reliable remedy, and as a result he publishes the following formula in the Cincinnati Lancet and Clinic. He claims that it will give entire satisfaction, if used as directed:

℞ Hydrastin.................................gr. ss
Boracic acid..................................gr. iss
Morph. acetate..............................gr. $\frac{1}{8}$
Chlor. sodium................................gr. j
Pure cocoa butter q. s. to make bougie three inches long.

The method of using it is, first have patient urinate, then cleanse the urethra with starch water, after which introduce bougie. Continue this morning and evening until the discharge ceases (which it does in from two to five days), and then one each evening for five evenings more.—*Med. and Surg. Rep.*

**Diabetes.**—Dr. Austin Flint, jr., adds four more cases of diabetes to the fifty two reported to the American Medical Association. The patients were placed on strict antidiabetic diet, and Clemens's solution of arsenite of bromine, beginning with three drops, increased to five, was also given. Of these four cases three were permanently relieved. In conclusion he adds: "Diabetes has become to-day a disease easily and certainly curable, provided that the treatment be not begun too late."—*Louisville Med. Times.*

**Can the Sex of the Child be Determined Before Birth.**—Dr. S. T. Lowry writes: Although cognizant of the fact that the pulse rate of the female infant is more rapid than that of the male, I had never, until recently, put the matter to a practical test in determining the sex of the child prior to birth. But noticing, in a recent journal, the statement that the sex can be so determined with almost unerring certainty, I resolved to put the matter to trial by careful examination and record of a sufficient number of cases to test the truth or falsity of the statement. Beginning with Nov. 1st, 1884, the following is my record for that month:

| Name. | Date. | No. of Labors. | Pulse. | Sex. |
|---|---|---|---|---|
| Mrs. R. | Nov. 1 | 3 | 124 | Male |
| " W. | " 6 | 2 | 140 | Female |
| " C. | " 14 | 4 | 144 | Female |
| " H. | " 15 | 2 | 120 | Male |
| " Y. | " 16 | 4 | 148 | Female |
| " W. | " 17 | 1 | 164 | " |
| " Q. | " 18 | 2 | 120 | Male |
| " C. | " 22 | 4 | 146 | Female |
| " R. | " 23 | 2 | 132 | " |
| " H. | " 26 | 2 | 128 | Male |
| " K. | " 27 | 4 | 148 | Female |
| " B. | " 28 | 3 | 122 | Male |
| " D. | " 29 | 2 | 120 | " |
| " L. | " 30 | 2 | 126 | " |

Now, examining the table just read it will be observed, that there are fourteen cases of confinement recorded, six of the infants born being girls, and eight boys. Now, by adding together the whole number of pulse beats per minute and dividing by 14 (the number of children) we have 133 for the average pulse beat of the infant at birth. Now, by examining the table again it will be observed that the pulse of every male child falls below this general average, while every female child rises above that number with the exception of the infant of Mrs. R., confined on the 23d of the month, who gave birth to a female child though the pulse was only 132. In this case, however, I ought, perhaps, to observe that the infant was quite large, weighing almost ten pounds.

From these few cases I think we are justified in concluding that a physician can, with a reasonable degree of accuracy, determine the sex of the fœtus at full term, and that if, at the close of the period of gestation in a normally developed fœtus the pulse rate is above 135, we we can with almost unerring certainty declare the child a female; while, on the other hand, if the beat is below that number, we can with almost certainty, declare the child a male. I should say, however, that a more extended record may change these figures to some extent.—*Texas Courier-Record.*

**An Unintentional Pun.**—Said a lady sympathiser to a Confederate hospital convalescent, (all old army surgeons can imagine the scene) "Do you keep a diary, my friend?" "Yes-um, I've had the dia-ree purty peart ever since I jined the army."

**The Cure of Asthma.**—From the *Medical Record*, Jan. 24, 1885:—I understand by the term asthma the condition of spasm of the bronchial tubes of both lungs, with hyperæmia approaching or amounting to inflammation, accompanied by rales upon both inspiration and expiration, with great difficulty of breathing. And the term is applied to the paroxysm alone, which returns at regular or irregular periods. Disturbance of function or disease of structure of the pneumogastric nerve is always present.

To cure the paroxysms I originated a method of treatment nearly five years ago; and repeated observation has confirmed its great utility. When called to a case of asthma, with a camel's hair brush I make a streak of Churchill's iodine *over each pneumogastric nerve, in its course in the neck, from the upper part of the thyroid cartilage to the upper borders of the clavicles.* By counter irritation thus applied, the capricious and abnormal exercises of nerve force by the pulmonary filaments is controlled, and bronchial spasm promptly relinquished. Such is my original method—simple, certain, quick. Churchill's tincture is the best counter-irritant, because, first, it is convenient; second, its action is easily controlled; third, it does the work. To permanently cure the paroxysms, it is usually necessary to remove the underlying morbid condition upon which they depend or are associated.—Richard B. Faulkner, M. D , *Quarterly Epitome.*

**Electricity in Obstetrics.**—Since October, 1883, Tipjakow has made use of a peculiar sound for the purpose of applying the electric current directly to the uterus. This consists of an elastic sound through which run two isolated wires whose extremities approach each other near the olive tip of the sound. After having reported in detail three observations he offers the following conclusions (*Deutsche Med. Zeit.*, Nov. 17, 1884):

1. The electric current is an energetic, simple and inoffensive means of stimulating uterine contraction. The indications for its use are the induction of premature labor, atony of the uterus and post-partum hemorrhage.

2. As the intensity of the contractions promotes the rapidity of dilatation of the neck, the faradic current may be employed to this end.

3. In the period following confinement, one can by the aid of electricity obtain more regular and complete involution of the uterus.

4. In cases of puerperal endometritis the faradic current acts by stimulating contractions and so causing the evacuation of the uterine cavity, which frees it more rapidly of the altered products of secretion.— *Weekly Medical Review.*

**Ergot in Treament of Dysentery.**—Twenty-one cases of dysentery in children, reported by Dr. G. L. Magruder, of Washington, were treated with fluid extract of ergot, five to twenty drops four or five times a day. Almost every case immediately responded to treatment, and was either entirely relieved or much improved.— *Va. Med. Reporter.*

**Hay Fever and its Successful Treatment.**—From the *N. Y. Med. Journal*, Dec. 6, 1884:—As a *resume* I would submit the following: That hay fever is an idiosyncrasy existing in certain individuals, to become influenced by certain emanations or irritating substances.

That the idiosyncrasy is accompanied by a chronic hyperæsthesia of that part of the nasal mucous membrane covering the inferior and middle and turbinated bones, the middle meatus, the floor of the nose, and that part of the septum below the limit of the olfactory membrane.

That organic alteration of those parts annuls that hyperæsthesia, preventing at the same time what symptoms the patient may be liable to in the course of an access.

That any destructive agent will induce that organic alteration, but that the galvanic cautery is by far the best, being painless, effective, and devoid of all danger when used in practiced hands.

That, in order to obtain a satisfactory result, a sufficient number of applications must be made, covering the entire extent of the over-sensitive surface, without which the result will be doubtful.

Now, gentlemen, permit me to state, in conclusion, that prior to last Saturday, and *after this paper had been written*, I knew nothing of Dr. Roe's (of Rochester) theory of hay fever, his method of treatment, and his results. The contribution I have just had the honor of presenting to you embodies precisely those views, and the results shown indicate evidently that the theory is the true one.—Charles E. Sajous, M. D., *Quarterly Epitome.*

**A New Treatment for Neuralgia.**—The latest agent introduced for the relief of neuralgia is a one per cent. solution of hyperosmic acid, administered by subcutaneous injection. It has been employed in Billroth's clinic in a few cases. One of the patients had been a martyr to sciatica for years, and had tried innumerable remedies, including the application of electricity no fewer than two hundred times, while for a whole year he had adopted vegetarianism. Billroth injected the above remedy between the tuber ischii and trochanter, and within a day or two the pain was greatly relieved, and eventually it quite disappeared. It would be rash to conclude too much from these results, in the face of intractibility of neuralgia to medication, but if it really proved to be as efficacious as considered, hyperosmic acid will be a therapeutic agent of no mean value.—*Ex.*

**Opium Habit.**—Dr. A. A. Post, of New York, thus describes his method of treating the opium habit: "A sudden reduction in the quantity of opium indulged in; not an immediate and total cessation of its use—which would be injurious—but a reduction in quantity covering a week or two weeks, and accompanied with stimulants of a different kind, such as hyoscyamus, belladonna, etc., until a cure is effected."

Under this method of treatment, he thought that every case not connected with a chronic painful affection could be cured. The after-treatment is similar to that pursued in cases of typhoid fever.

**Alvelos: A New Cure for Cancer.**—The plant commonly known by the name of alvelos belongs to the euphorbiacæ, and is indigenous to Pernambuco. Dr. Velloso, of that province, states, in a communication to the *Journal de Receife*, that a magistrate, who was suffering from epithelioma of the face, and who had returned to his estate despairing of relief, was entirely cured of his disease by the topical application of the juice of the plant. On the strength of this report Dr. Velloso tried the remedy in the case of cancroid of the nose, and in one of epithelioma of the lip, with the result that the first patient was completely cured in forty days, and the second in less than two months. These results, he thinks, justify a trial of the remedy, especially in the uterine cancer. The action of the juice of the plant is irritating, producing a spreading dermatitis without much pain, and the application of the cut stem or the juice of the fresh plant to the diseased part, is said to result in the destruction of the morbid tissue, which is replaced by healthy granulations, doing the work, in fact, of the chloride of zinc paste.—*New York Medical Times.*

**The Aspirator in Hydrothorax.**—Dr. E. D. Perguson, after recalling cases in which effusion from idiopathic pleurisy was completely removed by aspiration, only to suffer relapses which eventuated in pyo-thorax and death, warns against the operation, unless dyspnœa compels it or absorption is long delayed. If the object be to relieve dyspnœa, Dr. F. advises to remove just enough to relieve this condition, one pint being usually sufficient. If the object be to stimulate absorption he has found the removal of about eight ounces usually sufficient. Since adopting such a plan he has had no pyo-thorax follow. On the inflamed engorged pleura, with its exudate containing new-formed, thin-walled blood vessels, there exists air pressure from within and fluid pressure from without. To remove at one sitting all fluid pressure is to produce increased vascular supply, rupture of these new vessels, elements favoring suppuration.—*N. Y. M. Jour. St. Louis Med. Jour.*

**The Simaba Cedron as a Remedy for Hydrophobia.**—Vaillant ("Therap. Gaz.") recommends a preparation made from the seeds of this South American tree as a remedy for hydrophobia. The active principle is a bitter crystaline substance, which dissolves in hot water to form a neutral solution. The writer professes to have cured with it two cases of hydrophobia that had already reached an advanced stage of convulsions.—*New York Medical Journal.*

**Phenylhydrazine.**—Hoppe-Seyler ("Ztschr. f. phys. Chem."; "Ctrlbl. f. d. ges. Therap.") has experimented with this substance and its salts, and finds that, when introduced into the stomach of rabbits, or injected beneath the skin, they cause a rapid decomposition of the red-blood corpuscles and a setting free of the coloring matter. Very small doses are fatal, their administration being followed by hæmaturia and other symptoms of profound hæmic disturbance.—*New York Medical Journal.*

**Amblyopia.**—Dr. Gunn (in the Pacific Medical and Surgical Journal) says: In two cases of amblyopia, amounting to almost amaurosis, brought about by excessive use of alcohol and tobacco, hypodermic injections of strychnia were given, and the constant current was applied to the eyes and forehead, and the use of stimulants enjoined. One of the men who came regularly and gave up the use of the pipe and bottle, soon regained his sight fully. The other patient, who, probably, enjoyed his "pipe and toddy" better than electricity, soon ceased to come, though he had improved under treatment. From the benefit derived from this mode of treatment, in several cases of incipient atrophy of the optic nerve, I think that if a patient could have the injections and electricity three or four times a week the progress of the disease could, in many cases, be checked.

**Permanganate of Potassium in Amenorrhœa**, says an exchange, has been proved to be almost a specific. "Given in doses of from two to five grains three times a day, for several days preceding the menstrual period, this agent is quite sure to start the flow." The cases best suited for its administration are those characterized by torpor, anæmia, or deficient activity of the menstrual apparatus; it is contra-indicated where acute congestion or a general sthenic condition exists. From personal observation the Professor recommends it as a most effective remedy for obesity, its curative action being manifested without any aid from change of diet or exercise.

**Cannabis Indica as a Local Anæsthetic.**—A. Aaronson writes, in the British Journal of Dental Science, that tincture of cannabis indica as a local anæsthetic is perfectly satisfactory. He has extracted, with its aid, as many as twenty two teeth and stumps at one sitting. His plan is to dilute the tincture some three to five times, according to the probable duration of the operation. The diluted tincture is then applied in cotton-wool to cavities, if such exist, and also about the gums of the affected teeth. The beaks of the extracting forceps are also, after being warmed, dipped in the diluted tincture. In cold weather it is wise to dilute the cannabis indica.—*New York Med. Rec.*

**Jacaranda Lancifoliata.**—Mennell ("Brit. Med. Jour.") reports fourteen cases of venereal disease in which he used this drug with satisfactory results. Not only did he cure obstinate urethritis, but found it of decided benefit in two cases of secondary syphilis. For gonorrhœa he advises the use of the tincture, in doses of fifteen minims, together with injections of ten minims of the tincture to an ounce of water.—*Ibid.*

**The Muriate of Erythroxylon** (Cocain) relieves the turgescence of the venous sinuses incident to an ordinary cold. The relief lasts for from twelve to twenty-four hours. The remedy is used in the form of a spray. Bosworth calls attention to its value in this regard, and a considerable observation enables us to confirm his statements.—*Detroit Lancet.*

**Jarborandi in Obstinate Hiccough.**—Pagenstecher ("Ctrlbl. f. d. ges. Therap."; "Bull. gen. de Therap.") reports a case of hiccough which has resisted every known remedy, including the bromides, morphine, chloroform and electricity. The patient's diaphragm contracted in the most violent manner about twenty or thirty times a minute, and he had been unable to take any nourishment for three days. After receiving four grains of jaborandi-leaves, in the form of a decoction, he had a profuse perspiration, after which the hiccough was completely checked.—*N. Y. Med. Journal.*

**Twins Born Four Days Apart.**—Dr. James Douglas, of Morristown, N. J., writes: "A very unusual occurrence in the birth of twins has happened lately in my practice, one of the twins, a girl, being born at half past nine on Friday night, January 2, 1885 (breech presentation), and the other, a boy, on the following Tuesday afternoon at twenty minutes to three (head presentation), a difference of three days, seventeen hours and ten minutes. They are eight months children, the girl weighing four and one-half pounds, the boy six pounds. There was only one placenta. The mother is a healthy young woman, twenty-two years of age."—*N. Y. Med. Rec.*

**Reduction of Dislocation of the Humerus.**—Dr. Gissler's method of reduction:

1. The elbow is pressed against the abdomen and then gently drawn outward until resistance is met with.
2. The forearm is then raised as high as possible toward the opposite shoulder.
3. Then the whole arm is drawn outward, and the operation is finished.

This is a valuable addition to our knowledge of the operations which are daily needed. It is simple, accurate, and may be of use. —*South. Clinic.*

**The Treatment of Albuminuria with Chloral.**—Dr. Wilson states, in the British Medical Journal, that he has treated a few cases of albuminuria with this drug, and has noticed that by its constant use he was able to cause a complete disappearance of albumen from the urine, the albumen reappearing as soon as the remedy was suspended. The theory of its action is not stated.—*Med. Digest.*

**Perosmic Acid in Sciatica.**—Merces ("Lancet") has employed hypodermic injections of the acid in eighteen cases of sciatica, with complete success in twelve. From three to five drops of a one-per-cent. solution were injected deep in the region of the sciatic nerve, at a point midway between the tuber ischii and the trochanter major. The writer states that neither local nor general disturbance follows the injection. In some cases immediate relief was obtained after prolonged suffering.—*N. Y. Medical Journal.*

